# The Evaluation of Tumor Budding in Oral Squamous Cell Carcinoma Arising in the Background of Oral Submucous Fibrosis

**DOI:** 10.7759/cureus.69830

**Published:** 2024-09-21

**Authors:** Amba Esakki, Reshma Poothakulath Krishnan, Deepak Pandiar, Pratibha Ramani, Selvaraj Jayaraman

**Affiliations:** 1 Oral Pathology and Microbiology, Saveetha Dental College and Hospitals, Saveetha Institute of Medical and Technical Sciences, Saveetha University, Chennai, IND; 2 Centre of Molecular Medicine and Diagnostics (COMManD), Saveetha Dental College and Hospitals, Saveetha Institute of Medical and Technical Sciences, Saveetha University, Chennai, IND

**Keywords:** cancer, malignant transformation, oral squamous cell carcinoma, oral submucous fibrosis, tumor budding

## Abstract

Background and objective

Oral submucous fibrosis (OSMF) is a common and potentially malignant disorder with a high risk of malignant transformation to oral squamous cell carcinoma (OSCC). Tumor budding is a scattered pattern of invasion and is related to the aggressive behavior of malignant tumors, increased depth of invasion, higher clinical staging, size, and grade of the tumor. The present study aimed to evaluate tumor budding in OSCC arising in the background of OSMF.

Materials and methods

A total of 120 patients with OSCC (30 each of OSCC arising in the background of OSMF, well-differentiated, moderately differentiated, and poorly differentiated OSCC) were included in the study. Hematoxylin and eosin (H&E)-stained sections were evaluated for the presence of tumor buds at the invasive front of the tumor. Kappa statistics and chi-square tests were employed to statistically compare the results by using IBM SPSS Statistics 23 software (IBM Corp., Armonk, NY). A p-value of less than or equal to 0.05 was considered statistically significant.

Results

The mean age of the occurrence of OSCC arising in the background of OSMF was 45.3 ±7.62 years. A progressive increase in the tumor buds was noted in OSCC arising in the background of OSMF, well-differentiated squamous cell carcinoma (WDSCC), moderately differentiated squamous cell carcinoma (WDSCC), and poorly differentiated squamous cell carcinoma (PDSCC). The chi-square test showed no significant difference between OSCC in the setting of OSMF and WDSCC (p=0.604) groups; however, a significant difference was noted with MDSCC (p=0.001) and PDSCC (p=0.000) groups.

Conclusions

OSCC arising in the background of OSMF shows lower tumor budding at the invasive front of the tumor. This histopathological parameter can be easily identified in the H&E sections and is fairly reproducible. Hence, reporting the presence of tumor budding will help in predicting the prognosis of these patients.

## Introduction

Oral submucous fibrosis (OSMF) is one of the most common potentially malignant disorders of the oral cavity [1]. It is a commonly encountered lesion in South Asian countries due to the habit of arecanut chewing. The global prevalence of OSMF is 4.96% with a 95% CI: of 2.28-8.62 [2]. Arecoline in arecanut is one of the major causative agents for OSMF. The components of arecanut increase the high-risk alleles and genotypes of transforming growth factor beta 1 (TGF‐β1), matrix metalloproteinases (MMPs), and lysyl oxidase (LOX), leading to altered protein expression and causing collagen deposition [3]. This lesion shows fibrous bands causing blanching of oral mucosa with severe restriction of mouth opening. With disease progression, increased collagenization and homogenization are seen in the connective tissue, resulting in the loss of vascularity and replacement of muscle by fibrous connective tissue.

OSMF has a high potential for malignant transformation to oral squamous cell carcinoma (OSCC) [4]. The rate of malignant transformation is approximately 4.2% [2]. These tumors have different histopathological characteristics because of the activation of distinct molecular signaling pathways. A majority of the patients die from this disease because of local invasion, intravasation of tumors, and various proteolytic enzyme secretion [5]. The malignant transformation of OSMF is commonly seen in the buccal mucosa. Chronic inflammation of the mucosa and the reactive oxygen species and metabolites generated by arecanut contribute to malignant transformation. Most patients with OSCC arising from OSMF present at a very young age and are usually found to have well-differentiated squamous cell carcinomas (WDSCC) [6].

Tumor budding involves a scattered pattern of invasion with the presence of less than five cancer cells, in groups or individually at the invasive front of the tumor [7]. Tumor budding was initially described as “sprouting” by Imai et al. [8]. The cells in the tumor buds are less well-differentiated, with large nuclei showing loss of junctional complexes [9]. The tumor budding is related to the aggressive behavior of the tumor, increased recurrence rate, distant metastasis, and poor prognosis in numerous cancers like lung carcinomas, laryngeal carcinoma, and colorectal carcinoma including OSCCs [8,10,11]. Furthermore, increased tumor budding is associated with lymph node metastasis even in early OSCC. Tumor budding also strongly correlates with depth of invasion, clinical staging, size, and grade of the tumor. 

The present study aimed to evaluate the presence of tumor budding in OSCC arising in the background of OSMF. The evaluation of tumor budding will help in determining the prognosis of these patients. Furthermore, this can be assessed in hematoxylin and eosin (H&E)-stained sections and can be performed easily during routine pathological examination and the results are fairly reproducible.

## Materials and methods

Study design

This retrospective, cross-sectional study was conducted at a tertiary oral cancer center in Tamil Nadu, South India. Ethical committee approval was obtained from the institutional ethical committee (SRB/SDC/UG-2105/24/OPATH/191). A total of 120 patients with OSCC [30 OSCC arising in the background of OSMF, 30 WDSCC, 30 moderately differentiated squamous cell carcinoma (MDSCC), and 30 poorly differentiated squamous cell carcinoma (PDSCC)] were included in the study. The WDSCC, MDSCC, and PDSCC groups showed no clinical signs or histopathological features of OSMF. The inclusion criteria were as follows: cases of OSCC of any gender, aged more than 40 years with detailed clinicopathological details. Recurrent cases or patients who underwent chemoradiotherapy or any naturopathic treatment were excluded. Oral squamous cell carcinoma cases other than conventional OSCCs and those without H&E-stained slides or blocks were also excluded. H&E sections were retrieved and re-evaluated by two pathologists. New sections were made and H&E staining was performed if the stain had faded.

Evaluation of tumor budding

**Figure 1:**
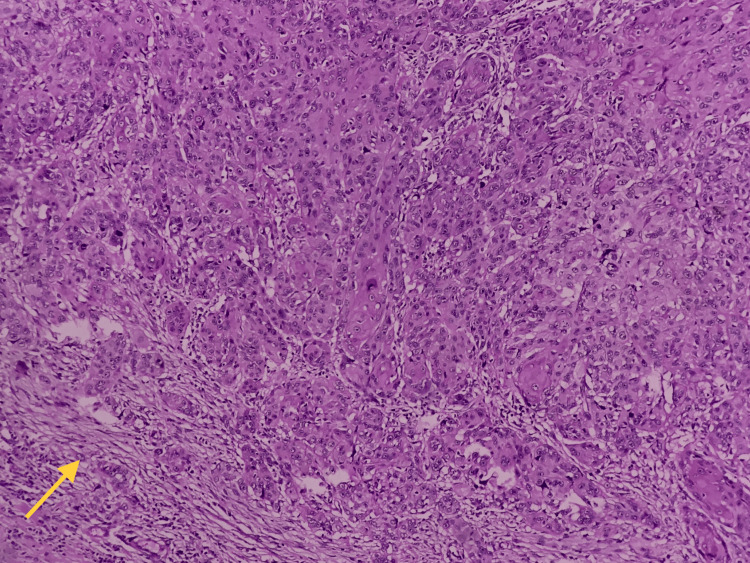
Photomicrograph of hematoxylin and eosin-stained section showing tumor buds at the invasive tumor front (100X)

Demographic and clinical details including age, gender, and site of the lesion were retrieved from the departmental records for these 120 patients. Two pathologists evaluated tumor budding at the invasive front of SCC. Both pathologists were blinded for the clinical data. The presence of 5 or <5 tumor cells in the invasive front were considered as tumor buds. The presence or absence of tumor buds was noted for all 120 OSCC cases (Figure 1).

The number of tumor buds present at the invasive tumor front was further evaluated and classified according to the revised International Tumor Budding Consensus Conference (ITBCC) guidelines [12]. The tumor buds were classified as follows: BO0, BD1, BD2, and BD3. BO0 represents no tumor buds, BD1: one to four tumor buds, BD2: five to nine tumor buds, and BD3: more than 10 tumor buds at the invasive tumor front.

Statistical analysis

The data were entered in Microsoft Excel and statistical analysis was performed using IBM SPSS Statistics 23 software (IBM Corp., Armonk, NY). WDSCC, MDSCC, and PDSCC were compared with OSCC arising from OSMF as well as with each other using a chi-square test. A p-value less than or equal to 0.05 was considered statistically significant. Inter-rater reliability was evaluated by Kappa statistics. Multiple regression analysis test was conducted on possible factors influencing tumor budding, including the gender, site, and different groups (OSCC arising in the background of OSMF, WDSCC, MDSCC, and PDSCC).

## Results

This study compared the clinicodemographic data and tumor budding between four groups: OSCC arising in the background of OSMF, WDSCC, MDSCC, and PDSCC cases. The mean age of occurrence of OSCC in the background of OSCC was 45.3 ±7.62 years. WDSCC patients had a mean age of 54.06 ±9.83 years, MDSCC: 51.91 ±7.3 years, and PDSCC: 53 ±10.5 years. It was noted that the age of occurrence of WDSCC was comparatively lower than in WDSCC, MDSCC, and PDSCC groups. The overall male-to-female ratio was 4:1 and most of the cases were reported in the buccal mucosa followed by gingivobuccal sulcus and lateral border of the tongue. OSCC arising in the background of OSMF was more common in males than females (13:2). A similar pattern was noted in the male-to-female ratio of WDSCC (23:7), MDSCC (19:11), and PDSCC (14:1). In terms of gender, the chi-square test showed no significant difference between OSCC arising in the background of the OSMF group and WDSCC (p=0.506), MDSCC (p=0.072), and PDSCC (p=0.671) groups (Table 1).

**Table 1:** Clinicodemographic details of included patients OSCC: oral squamous cell carcinoma; OSMF: oral submucous fibrosis; SCC: squamous cell carcinoma; SD: standard deviation

Parameter		OSCC arising in the background of OSMF	Well-differentiated SCC	Moderately differentiated SCC	Poorly differentiated SCC
Age, years, mean ±SD		45.3 ±7.62	54.06 ±9.83	51.91 ±7.3	53 ±10.5
Gender, n (%)	Male	26 (86.6%)	23 (76.6%)	19 (63.3%)	28 (93.3%)
	Female	4 (13.3%)	7 (23.3%)	11 (36.6%)	2 (6.6%)
Site, n (%)	Buccal mucosa	19 (63.3%)	11 (36.6%)	3 (10%)	12 (40%)
	Mandibular alveolus	2 (6.6%)	2 (6.6%)	8 (26.6%)	5 (16.6%)
	Gingivobuccal sulcus	5 (16.6%)	7 (23.3%)	7 (23.3%)	2 (6.6%)
	Lateral border of the tongue	3 (10%)	5 (16.6%)	9 (30%)	2 (6.6%)
	Hard palate	1 (3.3%)	0	0	1 (3.33%)
	Maxillary alveolus	0	2 (6.6%)	1 (3.33%)	3 (10%)
	Retromolar trigone	0	2 (6.6%)	1 (3.33%)	5 (16.6%)
	Floor of mouth	0	1 (3.33%)	1 (3.33%)	0

**Figure 2:**
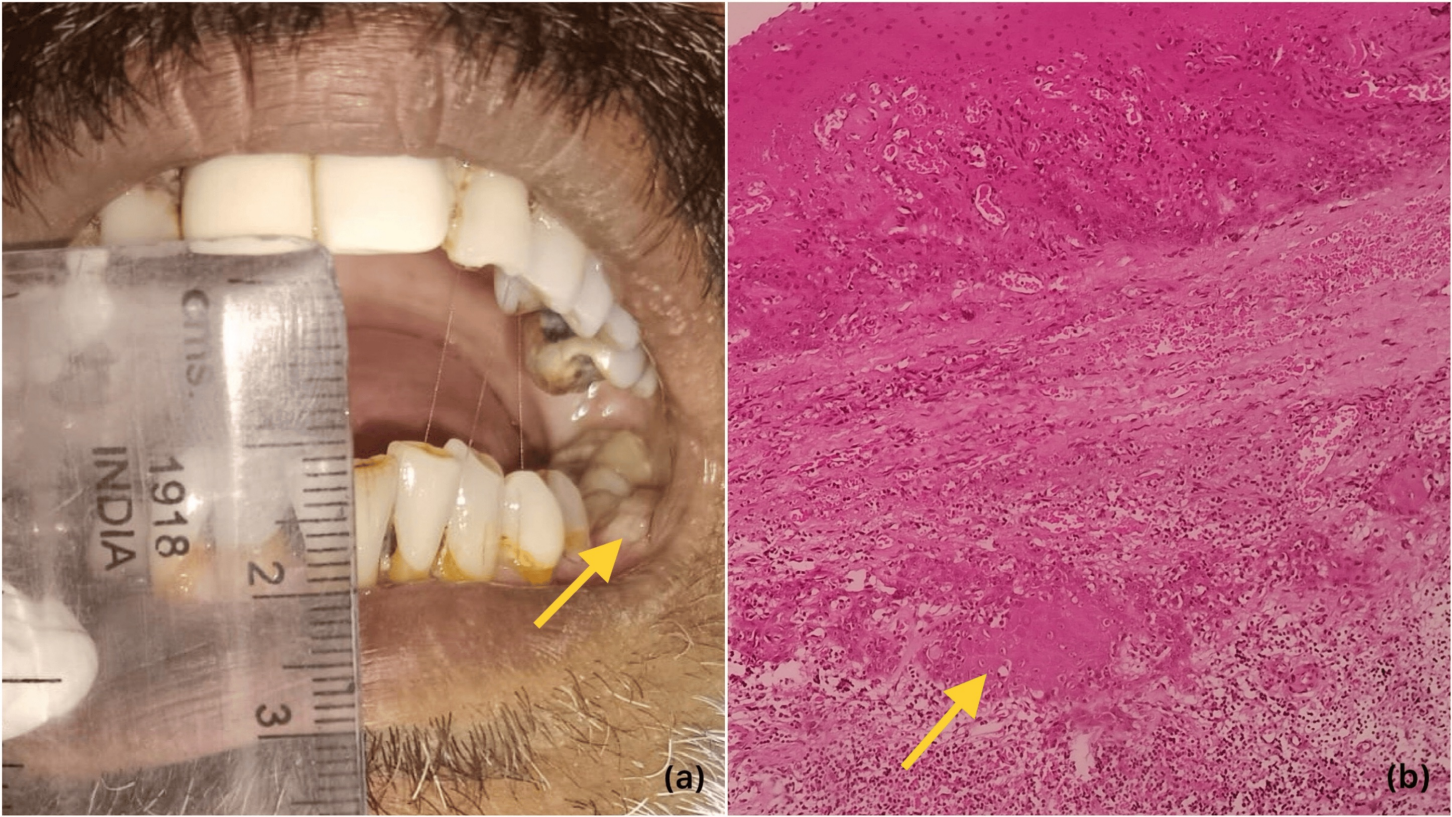
(A) clinical photograph of oral submucous fibrosis patient with oral SCC; the yellow arrow indicates the malignant lesion. (B) photomicrograph of tumor islands in collagenized stroma (H&E staining; 100X); the yellow arrow indicates the malignant tumor cells SCC: squamous cell carcinoma

The most common site of OSCC arising in the background of the OSMF group was buccal mucosa (n=19, 63.3%) followed by gingivobuccal sulcus (n=5, 16.6%), and lateral border of the tongue (n=3, 10%). The WDSCC and PDSCC were also common in the buccal mucosa (n=11, 36.6% and n=12, 40% respectively). The most common site for MDSCC was the lateral border of the tongue (n=9, 30%). No significant difference was noted between OSCC arising in the background of the OSMF group and WDSCC (p=0.255), while a significant difference was noted with MDSCC (p=0.002) and PDSCC (p=0.05) groups. Furthermore, most OSCCs arising in the background were well-differentiated carcinomas (Figure 2).

**Figure 3:**
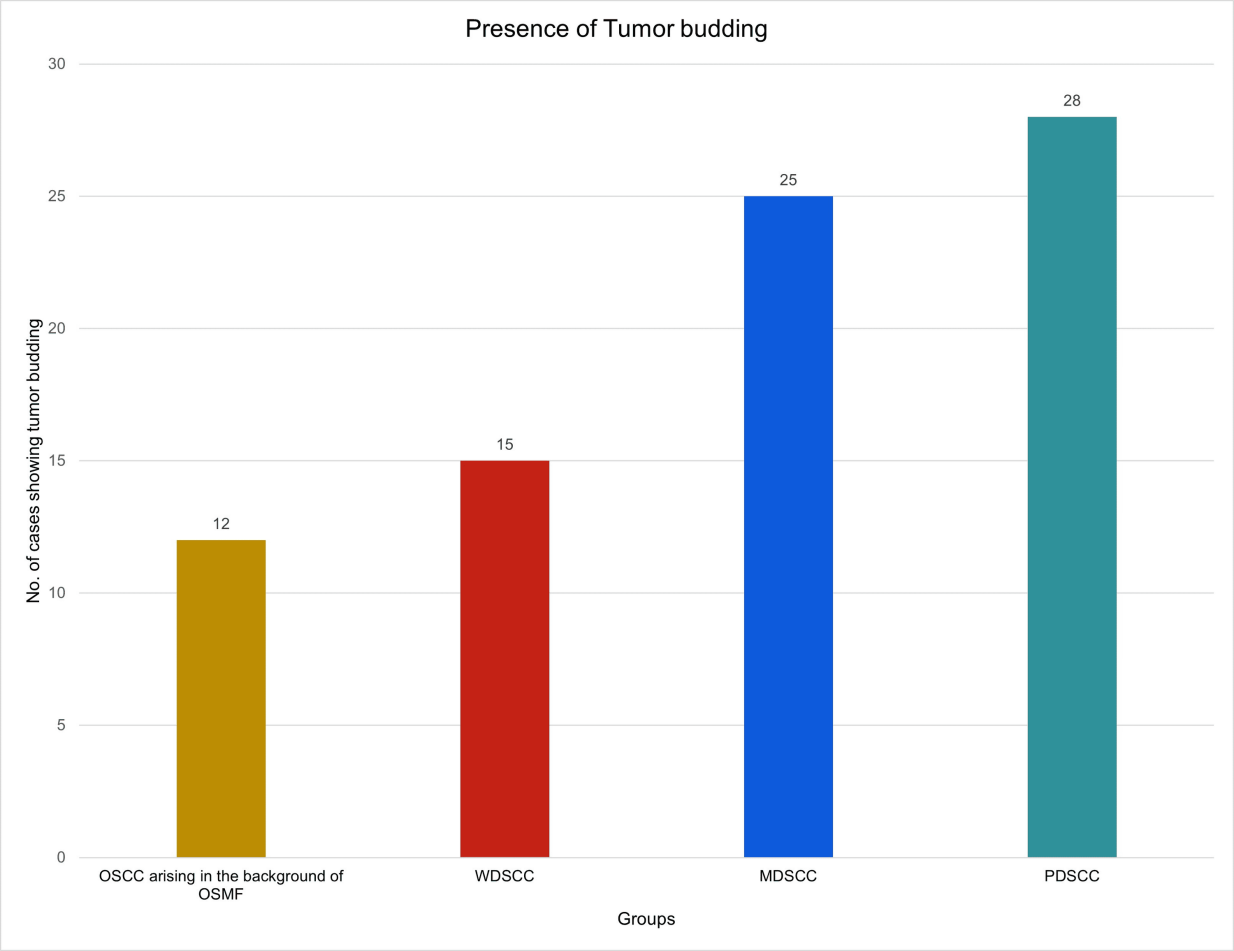
Progressive increase in the tumor budding was noted from OSCC arising in the background of OSMF to WDSCC, MDSCC, and PDSCC groups MDSCC: moderately differentiated squamous cell carcinoma; OSCC: oral squamous cell carcinoma; OSMF: oral submucous fibrosis; PDSCC: poorly differentiated squamous cell carcinoma; WDSCC: well-differentiated squamous cell carcinoma

The presence or absence of tumor budding was evaluated at the invasive front of the tumor. Tumor budding was noted in 12 cases (40%) of OSCC arising in the background of OSMF, 15 cases (50%) of WDSCC, 25 cases (83.3%) of MDSCC, and 28 cases (93.3%) of PDSCC groups. A progressive increase in the tumor buds was noted in OSCC arising in the background of OSMF, WDSCC, MDSCC, and PDSCC (Figure 3).

The chi-square test showed no significant difference between OSCC in the background in the OSMF and WDSCC (p=0.604) group, while a significant difference was noted with the MDSCC (p=0.001) and PDSCC (p=0.00) groups. When tumor budding was correlated with gender and site of OSCC - OSMF patients, no significant difference was noted either with respect to gender (p=0.469) or site (p=0.552). However, increased tumor budding was noted in male patients. Regarding the site, buccal mucosa showed increased tumor budding followed by gingivobuccal sulcus and lateral border of the tongue.

When the number of tumor buds at the invasive tumor was compared between the different groups, it was noted that no tumor buds (BD0) were reported in 18 (60%) cases of OSCC arising in the background of OSMF, 15 (50%) cases of WDSCC, five (16.6%) cases of MDSCC, and two (6.66%) cases of the PDSCC group. One to four tumor buds (BD1) were seen in 11 (36.6%) cases of OSCC arising in the background of OSMF and WDSCC groups, six (20%) cases of MDSCC, and three (10%) of PDSCC cases. Only one (3.3%) case of OSCC arising in the background of OSMF, four (13.3%) cases of WDSCC, 14 (46.6%) cases of MDSCC, and 10 (33.3%) of PDSCC cases showed five to seven tumor buds (BD2). More than 10 tumor buds (BD3) were noted in five (16.6%) cases of MDSCC and 15 (50%) cases of PDSCC group. BD3 were not seen in OSCC arising in the background of OSMF and WDSCC groups. When the number of tumor buds was compared between the four groups, the p-value obtained was 0.00 (statistically significant).

The results of the multiple regression analysis showed that different groups (OSCC arising in the background of OSMF, WDSCC, MDSCC, and PDSCC) were independent factors influencing tumor budding (p=0.00). The gender and site of the lesion showed a p-value of 0.667 and 0.885 respectively; indicating that these two factors had no independent effect on tumor budding.

## Discussion

The prognosis and the treatment outcome of OSCC depend on various factors. Recently, researchers have been focussing on the role of tumor microenvironment in tumor progression. Tumor budding is considered an independent prognostic factor for various tumors including OSCC [8]. Tumor buds characterize the aggressiveness and invasiveness of the tumors. These cells lose their cellular adhesion, increase the adjacent stromal degeneration, and resist apoptosis to increase their invasive ability [12]. This study evaluated the presence of tumor budding in OSCC arising in the background of OSMF. OSMF has an increased potential for malignant transformation to OSCC. These patients are known to have distinct molecular pathways and different histopathology, morphology, and pathogenesis. Our study noted that the mean age of patients reported with OSCC in the background of OSCC was 45.3 years. Similar results have been reported by Rangaswamy et al., who reported a mean age of 44.5 years for these patients [13]. A recent meta-analysis reported the average age of patients with OSCC from OSMF as 48.78 years [14].

Arecanut, an important etiological factor for OSMF, is a Class I carcinogen and might contribute to this malignant transformation [15]. The early and long-term exposure to tobacco products from a younger age followed by the development of early OSMF accelerates the malignant transformation. Our study showed an increased preponderance of OSCC arising from OSMF in males. This could be attributed to the increased incidence of tobacco and arecanut chewing in males. However, contradictory results have been reported from Pakistan; Mohiuddin et al. reported an increased incidence in females when compared to males [16]. These authors documented increased betel nut consumption and more toxic ingredients in young females [16]. 

In the present study, the buccal mucosa followed by the gingivobuccal sulcus and tongue were the most common sites for patients with OSCC arising from OSMF. Rangaswamy et al. observed that 28 out of 30 cases included in their study reported a lesion in the gingivobuccal complex [13]. Wang et al. evaluated 5071 patients and showed that 53.43% of cases were reported in the buccal mucosa [17]. Possibly, the chronic mucosal inflammation seen in the buccal mucosa due to trauma, and the components of the arecanut trigger the malignant transformation in this region. Furthermore, chronic inflammation exposes the deeper mucosal tissue to carcinogens, contributing to the carcinogenesis [6]. Loss of papillae of the tongue is a common clinical presentation in OSMF patients. This leads to the loss of the protective barrier of the tongue, further predisposing them to cancer development [16].

The invasive front of the tumor represents the area of active invasion and constant cross-talk between the cancer cells and the tumor microenvironment. Tumor budding is an important constituent of the tumor microenvironment. The tumor budding in cancers represents cells undergoing epithelial-mesenchymal transition (EMT) [18]. These cells show reduced immunohistochemical expression of E-cadherin and increased expression of vimentin [19]. A higher expression of laminin-5 gamma 2 chain and myofibroblasts is reported in the surrounding connective tissue stroma around these buds [20]. Decreased miR-200c, miR-200a, miR-200b expression is also noted in the tumor cells [21].

Evidence from the literature also suggests the presence of cancer stem cells in these tumor buds [22]. The present study noted that increased tumor budding in OSCC arising in the background of the OSMF group was seen in the buccal mucosa, and lesser tumor buds were seen in the OSCC arising from the tongue. The OSCCs arising from buccal mucosa are aggressive tumors with increased depth of invasion. This can be attributed to the increased tumor budding in this region. The increased incidence of tumor budding in OSCC-OSMF of buccal mucosa could also be attributed to the absence of a clear anatomical barrier in the buccal mucosa and its close proximity with the buccal pad of fat. The muscle fibers of the tongue and the presence of increased collagenization and hyalinization seen in OSMF possibly prevent the formation of tumor buds in the tongue.

Our results showed that OSCC arising from OSMF showed less tumor budding when compared to WDSCC, MDSCC, and PDSCC groups. Furthermore, it was also noted that OSCC arising in the background of OSMF were well-differentiated tumors. This could be attributed to the increased epithelial turnover and the possibility of genetic memory retained by these cells for differentiation. Furthermore, the fibrotic bands in OSMF prevent the infiltration of the tumor cells to deeper connective tissue stroma [23]. Sarode et al. suggested that basal cell hyperplasia, high proliferative activity of OSMF, and fibrosis in and around the minor salivary glands cause genetic alterations and faster differentiation of keratinocytes, leading to a better differentiation of oral squamous cell carcinoma [23].

Various studies in the literature have observed that WDSCC shows fewer tumor buds than MDSCC and PDSCCs [22]. These studies have also shown a significant association between tumor buds and poor overall survival in OSCCs. Furthermore, Almangush et al. observed that TB can be used to stratify malignancies into high-risk and low-risk cancers [24]. The fewer tumor buds in OSCC arising in the background of OSMF further support that these are less aggressive tumors. Gadbail et al. reported that OSMF-OSCC tumors show a lower incidence of nodal metastasis, less tumor thickness, and smaller tumor size [25]. As tumor budding plays an important role in these characteristics, the low tumor budding in OSCC arising in the background of OSMF prevents the tumor invasion deep into the connective tissue stroma, neoplastic dissemination, and lower chance of metastasis.

Furthermore, the hyalinization and collagenization in OSMF possibly prevent the dissociation of cells in the invasive front of the tumor. Hyalinization in oral lesions is known to influence the biological behavior of the tumor and is viewed as a mechanism to wall off the invasion of malignant cells [26]. Downregulation of the inhibitory effect of SMAD7 and SMAD6 proteins along with decreased degradation of collagen leads to the formation of hyalinized connective tissue stroma in OSMF [26]. Furthermore, the fewer tumor buds and blockage of submucosal lymphatics reduce the possibility of lymphatic spread in these patients. The hyalinization and collagen deposition in OSMF possibly decrease the vascularity and cause a hypoxic microenvironment. This decreases the nutrient supply to the tumor cells, further reducing the invasion and tumor bud formation. 

This study has a few limitations. The sample size was relatively lower owing to time constraints. Furthermore, additional immunohistochemical and molecular characterization, including the evaluation of confounding factors, could not be performed. We recommend further studies with larger sizes to address these limitations and gain deeper insights.

## Conclusions

Our findings show that OSCCs arising in the background of OSMF are commonly seen in younger males, with buccal mucosa being the most common site. These lesions show lesser tumor budding at the invasive front of the tumor. Tumor buds can be easily identified in the H&E sections and are fairly reproducible. As tumor budding reflects the biological behavior of the tumor, the presence of tumor buds should be mentioned in the pathology reports as these will help in predicting the prognosis of these patients. Further studies with larger sample sizes and molecular analysis are recommended to evaluate the pathogenicity of these tumor buds. In addition, understanding the pathophysiology and genetic background of tumor budding can aid in the identification of treatment targets for OSCC arising in the background of OSMF.
